# An Evaluation of Minimal F-wave Sensitivity in Carpal Tunnel Syndrome

**DOI:** 10.7759/cureus.60964

**Published:** 2024-05-23

**Authors:** Fahrettin Ege, Memet Aslanyavrusu

**Affiliations:** 1 Neurology, Yüksek İhtisas University, Ankara, TUR; 2 Neurology, Kayseri City Hospital, Ankara, TUR

**Keywords:** electrophysiology, entrapment neuropathy, hand, f-wave, median nerve, carpal tunnel syndrome

## Abstract

Background and objective

Several recent studies have explored whether F-waves can be a sensitive and useful tool for diagnosing carpal tunnel syndrome (CTS). In light of this, we aimed to measure the minimal F-wave latencies in patients with mild, moderate, and severe electrophysiologically diagnosed carpal CTS, as well as individuals without CTS, to determine at which point this parameter becomes sensitive to the syndrome.

Materials and methods

Nerve conduction studies were carried out in a room where a temperature of 22-24 °C was maintained. The F-waves of the median nerve in all patients and individuals in the control group were recorded. The F-wave with the highest velocity (minimal F) was categorized into the normal, mild, moderate, and severe groups for statistical analysis. All measurements were performed by the same electrophysiology-experienced neurologist.

Results

Post-hoc analysis demonstrated that the F latency values of the moderate and severe CTS groups were significantly higher than those of the control group (p<0.001 for all comparisons). Furthermore, the F latency values of the severe CTS group were significantly higher than those of the moderate group (p=0.026).

Conclusions

Based on our findings, minimal F-wave latency is a reliable indicator of moderate to severe CTS. This sensitivity significantly increases in severe cases while disappearing in the presence of mild CTS.

## Introduction

Carpal tunnel syndrome (CTS) is the most prevalent entrapment neuropathy globally and is observed in approximately 3.8% of people [1]. The disease and its treatment impose a heavy financial burden and can significantly impact patients' quality of life. Researchers and clinicians are still exploring the optimal treatment options for this condition. Currently, in addition to surgical methods, physical therapy is widely employed in the management of CTS and is effective for pain, physical function, and nerve conduction disorders [2].

F-waves are motor fiber action potentials obtained by antidromic stimulation with a bipolar electrical stimulator. They are late muscle responses and result from the activation of the anterior horn cells [3]. The measurement of F-wave latency is crucial for the electrophysiological diagnosis of demyelinating polyneuropathies, including Guillain-Barré syndrome (GBS) and chronic inflammatory demyelinating polyradiculoneuropathy (CIDP) [4]. It has also been used in the diagnosis of radiculopathies caused by factors such as disc compression and trauma, and anterior horn cell damages like poliomyelitis [5-7]. Electrophysiological diagnosis, however, is subject to disease vulnerability.

In recent years, many studies have explored whether F-waves can be a sensitive and useful tool for diagnosing CTS. For example, a study found that F-wave parameters were more sensitive in detecting CTS compared to the control group, suggesting that F-wave studies should be included alongside traditional electrophysiological methods in its diagnosis [8]. Some studies have suggested that electrophysiological values are not as sensitive in detecting CTS when examining F-wave parameters [9,10]. Therefore, our objective in this study is to measure the minimal F-wave latencies in patients with mild, moderate, and severe electrophysiologically diagnosed CTS, as well as individuals without CTS, to determine when this parameter becomes sensitive to the syndrome. We aim to accomplish this through a comprehensive study of a large group of participants.

## Materials and methods

Study design and subjects

Informed consent was obtained from all the participating patients and controls, and the study was approved by the 2nd Ethics Committee of Ankara Bilkent City Hospital (approval no: E2-23-4736). The study was conducted at the Electrophysiology Laboratory of VM Medicalpark Ankara Hospital from June to December 2023. The CTS sample exclusively comprised individuals between 160-185 cm in height, who were referred from various polyclinics including Neurology, Brain Surgery, Physical Medicine and Rehabilitation, Orthopaedics, and Internal Medicine with a preliminary diagnosis of CTS. Patients for whom EMG was requested with a preliminary diagnosis of CTS but whose electrophysiology was normal were excluded from the study. The study also excluded individuals with demyelinating or axonal polyneuropathy of any origin, a history of cervical vertebra trauma or surgical intervention, and those with traumatic nerve damage in the upper and forearm. After the examinations, participants were divided into three categories: "mild CTS," "moderate CTS," and "severe CTS." Volunteers being monitored for migraine in the neurology outpatient clinic, who had no complaints of pain, numbness, paraesthesia, or discomfort in their hands and had normal electrophysiological findings were randomly included in the control group.

Electrophysiology 

Nerve conduction studies were carried out in a room where a temperature of 22-24 °C was maintained. Skin temperature was maintained at 30-32 degrees. The bipolar recording electrode for the median nerve motor conduction study was positioned on the abductor pollicis brevis muscle, with supramaximal stimulation applied at two points - the wrist and elbow. The examination involved the measurement of distal latency, motor conduction velocity, and amplitude of compound muscle action potential (CMAP). During sensory conduction investigation, recording electrodes were placed on the median nerve at the wrist level, and the orthodromic action potentials that ensued were measured from the second finger and palm sections. This process facilitated recordings of sensory conduction velocities and action potential amplitudes.

For the F-wave study, the recording electrode was placed on the abductor pollicis brevis muscle and the median nerve was antidromically stimulated with 10 consecutive wrist stimuli. Volunteers included in the control group and whose electrophysiology did not show any abnormalities were evaluated in the normal category. Those with normal motor conduction studies but slowed sensory conduction and/or reduced sensory action potential amplitudes were classified as "mild CTS". “Moderate CTS” was associated with the prolongation of motor distal latency and abnormal sensory velocities and/or amplitudes. Patients were classified as having "severe CTS" if they had a prolonged distal latency and had decreased CMAP amplitude, abnormal sensory conduction study results, or absence of sensory action potentials. The F-waves of the median nerve in all patients and controls were recorded. The F-wave with the highest velocity (minimal F) was categorized into the normal, mild, moderate, and severe groups for statistical analysis.

All measurements were executed by the same electrophysiology-experienced neurologist. Each participant in both the patient and control groups provided one hand for statistical analysis. Participants with normal electrophysiological parameters in both hands were included in the control group and their dominant hand was evaluated. Participants with the one hand with nerve damage were included in the appropriate subgroup of the patient group. If a participant had CTS in both hands, the more severely affected hand was assigned to the relevant subgroup, and the other hand was excluded from the study. This methodology intended to exclude personal factors like height and cervical degeneration that could affect the outcomes. Accordingly, the study recorded and included electrophysiological parameters of only one hand per participant.

Statistical analysis

SPSS Statistics version 22 (IBM Corp., Armonk, NY) was used for statistical analyses. Absolute frequencies (n) and percentages (%) were used for reporting descriptive statistics for categorical data. The mean ± standard deviation (SD) was calculated and documented for numerical data with normal distribution. The Chi-square test was utilized for comparing proportions among categorical variables. The normal distribution assumption for numerical data was evaluated using the Shapiro-Wilk test, histograms, and Q-Q plots. We further assessed the homogeneity of variances using Levene's test. To compare numerical data that follows a normal distribution across three independent groups, we utilized a one-way analysis of variance (ANOVA). Post-hoc tests, either Tukey or Games-Howell, were selected based on the assumption of homogeneity of variances to ascertain groups with noteworthy disparities after the ANOVA test.

A receiver operating characteristic (ROC) analysis was conducted to assess the potential of F latency values in predicting CTS. ROC curves and the area under the curve (AUC), along with 95% confidence intervals (CIs), were computed and used to classify the AUC values as excellent (0.9-1), good (0.8-0.9), fair (0.7-0.8), weak (0.6-0.7), or poor (0.5-0.6). To establish the optimum cut-off point during ROC analysis, we utilized the Youden Index, which maximizes both sensitivity and specificity. We evaluated the cut-off point performance by taking into account sensitivity, specificity, positive predictive value (PPV), negative predictive value (NPV), and positive likelihood ratio (LR+) values. Additionally, we carried out univariate and multivariate binary logistic regression analyses to assess the influence of F latency values on CTS. Odds ratios (OR) were assigned to each statistically significant parameter in both univariate and multivariate models, accompanied by 95% CIs. In all comparisons, a level of p<0.05 was set as the threshold for statistical significance.

## Results

The study analyzed data from 173 volunteers divided into four groups: the control/normal group (27.7%, n=48), mild CTS group (25.4%, n=44), moderate CTS group (26.6%, n=46), and severe CTS group (20.2%, n=35). The mean age of all volunteers was 47.54 ±13.81 years (range: 18-75 years). Statistical outcomes for the comparison of gender, age, and F latency values among research groups are reported in Table 1. The gender ratio distribution among the groups varied significantly (p<0.001, Table 1). Additionally, the mean ages differed significantly (p=0.011, Table 1). Further analysis showed that the severe CTS group had a significantly higher mean age compared to the control group (p=0.005). However, no significant age differences were found between the other groups (p>0.05, Table 1). However, F latency values displayed a statistically significant difference between the control and CTS groups (p<0.001, Table 1).

**Table 1:** Statistical findings for comparison of gender, age, and F latency values among study groups ^a^Chi-square test with n (%). ^b^One-way ANOVA with mean ±standard deviation (SD). ^c^Tukey post-hoc test following one-way ANOVA. ^d^Games-Howell post-hoc test following one-way ANOVA

Findings	Normal (1) (n=48)	Mild (2) (n=44)		Moderate (3) (n=46)		Severe (4) (n=35)		P-value		Post-hoc p-value	
Gender	19 (39.6%) (male)	4 (9.1%) (male)		5 (10.9%) (male)		2 (5.7%) (male)		<0.001^a^			
	29 (60.4%) (female)	40 (90.9%) (female)		41 (89.1%) (female)		33 (94.3%) (female)					
Age (years)	43.23 ±12.97	47.64 ±13.12		47.48 ±12.83		53.40 ±15.36		0.011^b^		1-2: 0.401^c^	
										1-3: 0.424^c^	
										1-4: 0.005^c^	
										2-3: 1.000^c^	
										2-4: 0.238^c^	
										3-4: 0.209^c^	
F latency	24.9 ±1.58	25.02 ±1.21		27.38 ±2.14		29.64 ±4.16		<0.001^b^		1-2: 0.978^d^	
										1-3: <0.001^d^	
										1-4: <0.001^d^	
										2-3: <0.001^d^	
										2-4: <0.001^d^	
										3-4: 0.026^d^	

**Figure 1:**
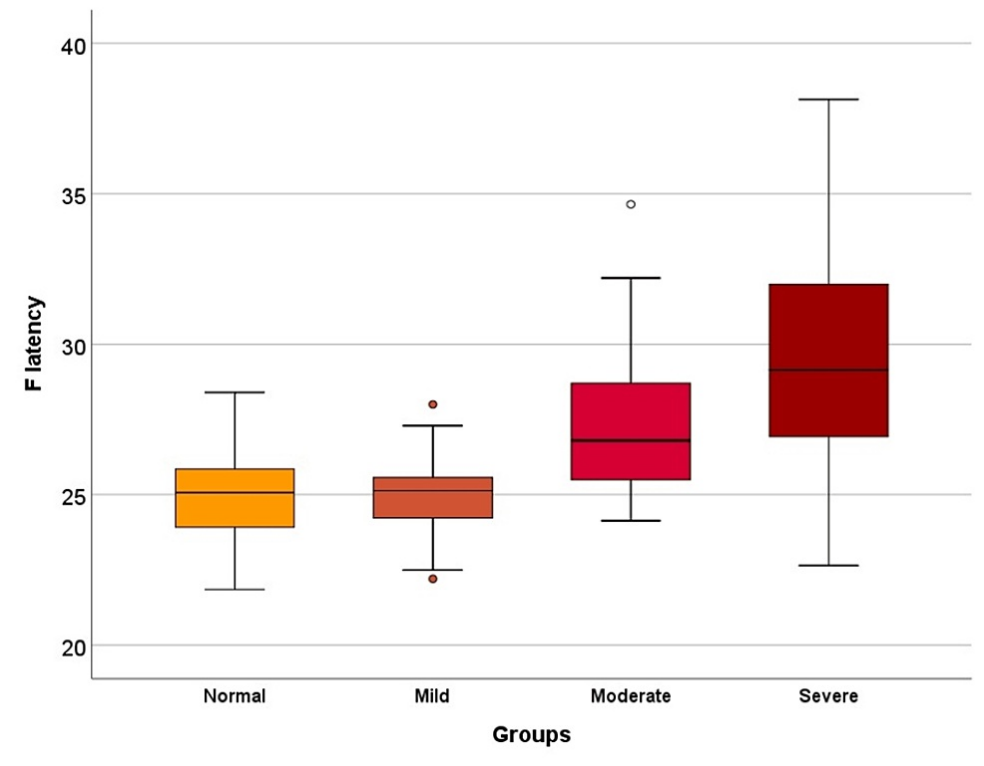
Box plot showing the distribution of F latency values among study groups

Post-hoc analysis demonstrated that the F latency values of the moderate and severe CTS groups were significantly higher than those of the control group (p<0.001 for all comparisons). Furthermore, the F latency values of the severe CTS group were significantly higher than those of the moderate group (p=0.026). However, no significant difference was observed in F latency values between the control and mild CTS groups (p=0.978) (Figure 1).

Table 2 displays the results of the ROC analysis, which aimed to assess the predictive success of F latency in CTS. The analysis also calculated sensitivity, specificity, PPV, and NPV using the cut-off values established by ROC analysis. Two group differentiations were analyzed. The first analysis distinguished the CTS group from the control group. Since no significant difference was observed in F latency values between the control and mild CTS groups, a secondary ROC analysis was conducted to distinguish the control + mild CTS groups from the moderate + severe CTS groups.

**Table 2:** Findings of the ROC analysis with sensitivity, specificity, PPV-NPV, and positive LR values that demonstrate the success of F latency values in predicting CTS CTS: carpal tunnel syndrome; ROC: receiver operating characteristic; AUC: area under the curve; PPV: positive predictive value; NPV: negative predictive value; LR: likelihood ratio; CI: confidence interval

Findings	F latency (normal vs. CTS)	F latency (normal + CTS mild vs. CTS moderate + severe)
AUC (95% CI)	0.727 (0.650-0.804)	0.851 (0.794-0.909)
P-value	<0.001	<0.001
Cut-off	26.575	26.625
Sensitivity (95% CI)	48% (39-57.1)	66.7% (55.2-76.5)
Specificity (95% CI)	89.6% (76.6-96.1)	89.1% (80.5-94.4)
PPV (95% CI)	92.3% (82.2-97.1)	84.4% (72.7-91.9)
NPV (95% CI)	39.8% (30.7-49.7)	75.2% (65.9-82.8)
LR + (95% CI)	4.61 (1.97-10.8)	6.13 (3.35-11.2)

**Figure 2:**
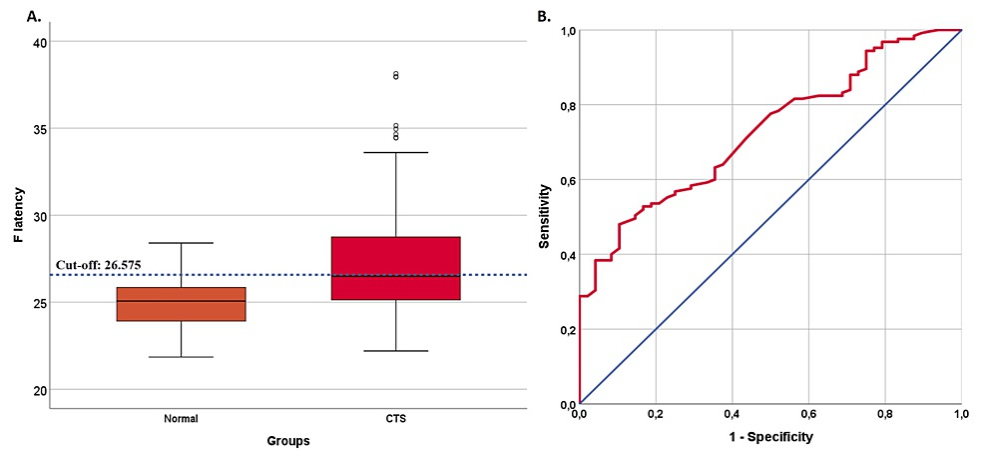
A boxplot (A.) showing the distribution of F latency values and the optimal cut-off point in the differentiation between the normal group and CTS groups, along with the ROC curve (B.) indicating the success of F latency in group differentiation CTS: carpal tunnel syndrome; ROC: receiver operating characteristic

**Figure 3:**
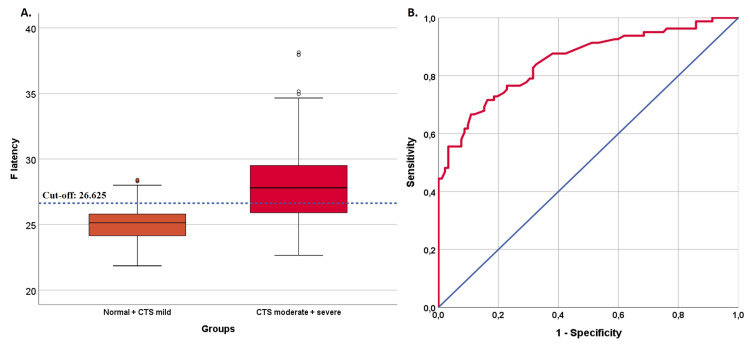
A boxplot (A.) showing the distribution of F latency values and the optimal cut-off point in the differentiation between the normal + CTS mild groups and CTS moderate + severe groups, along with the ROC curve (B.) indicating the success of F latency in group differentiation CTS: carpal tunnel syndrome; ROC: receiver operating characteristic

The ROC analysis results demonstrate that F latency values effectively differentiate the control group from the CTS groups (AUC=0.727, see Table 2). The optimized F latency cut-off point was calculated at 26.575, with a sensitivity of 48% (39-57.1), specificity of 89.6% (76.6-96.1), PPV of 92.3% (82.2-97.1), and NPV of 39.8% (30.7-49.7). Figure 2 depicts a box plot representing the distribution of F latency values, demonstrating the most successful cut-off point, alongside an ROC curve that illustrated the performance of F latency in differentiating between the control group and CTS groups. Notably, F latency values showed significant differences in distinguishing between the control + mild CTS group and the moderate + severe CTS groups (AUC=0.851, Table 2). The most effective cut-off point determined for F latency was 26.625, exhibiting a sensitivity of 66.7% (55.2-76.5), specificity of 89.1% (80.5-94.4), PPV of 84.4% (72.7-91.9), and NPV of 75.2% (65.9-82.8). Figure 3 displays a boxplot of F latency values with the optimal cut-off point and an ROC curve that demonstrates the proficiency of F latency in distinguishing between the control + mild CTS group and the moderate + severe CTS groups.

The results of univariate and multivariate binary logistic regression analyses to assess the impact of F latency groups on CTS, while using ROC analysis determined cut-off points, are presented in Tables 3-4. In the univariate model performed to differentiate the control group from CTS ones, age, gender, and F latency significantly influenced the results (p=0.012, p<0.001, p<0.001, respectively; Table 3). In the univariate model, the OR for F latency was 7.94 (2.95-21.37). In the multivariate model with age, gender, and F latency, the effect of age was not significant (p=0.698). Consequently, in the final model, established with F latency and gender only, the OR for F latency was 9.43 (3.21-27.7), and the OR for gender was 8.34 (3.13-22.2). Patients with an F latency value greater than 26.575 had a 9.43 times greater chance of developing CTS than those with a lower value.

**Table 3:** Results of univariate and multivariate binary logistic regression analyses performed to determine the effect of F latency values on CTS (in the differentiation of normal and CTS groups) Multivariate model: (gender + F latency) Nagelkerke R squared=0.327. Classification accuracy: 78.6% Values below p<0.05 are shown in bold NS: not significant; OR: odds ratio; CI: confidence interval; CTS: carpal tunnel syndrome

Findings	Univariate		Multivariate	
	P-value	OR (95% CI)	P-value	OR (95% CI)
Age (years)	0.012	1.034 (1.007-1.061)	NS	-
Gender (Female and male)	<0.001	6.79 (2.91-15.84)	<0.001	8.34 (3.13-22.2)
F latency (≥26.575 and <26.575)	<0.001	7.94 (2.95-21.37)	<0.001	9.43 (3.21-27.7)

**Table 4:** Results of univariate and multivariate binary logistic regression analyses performed to determine the effect of F latency values on the severity of CTS (in the differentiation of normal + mild groups and CTS moderate + severe groups) Multivariate model: (gender + F latency) Nagelkerke R squared=0.449. Classification accuracy: 78.6% Values below p<0.05 are shown in bold NS: not significant; OR: odds ratio; CI: confidence interval; CTS: carpal tunnel syndrome

Findings	Univariate		Multivariate	
	P-value	OR (95% CI)	P-value	OR (95% CI)
Age (years)	0.027	1.026 (1.003-1.049)	NS	-
Gender (female and male)	0.007	3.52 (1.42-8.73)	0.005	5.55 (1.67-18.4)
F latency (≥26.625 and <26.625)	<0.001	16.4 (7.35-36.6)	<0.001	20.5 (8.22-51.2)

When differentiating the control + mild CTS group from the moderate + severe CTS groups, the effects of age, gender, and F latency were significant (P=0.027, P=0.007, P<0.001, respectively, refer to Table 4). In the Univariate model used, the odds ratio (OR) for F latency was 16.4 (7.35-36.6). In the multivariable model carried out with age, gender, and F latency, age did not have a significant effect (P=0.491). Consequently, in the final model determined with F latency and gender, the odds ratio (OR) for F latency was 20.5 (8.22 - 51.2), and the OR for gender was 5.55 (1.67 - 18.4). The likelihood of developing CTS in patients with an F latency value higher than 26.625 was 20.5 times greater than in those with an F latency value less than 26.625.

## Discussion

Our research validates the findings of earlier studies that there is a greater prevalence of CTS among women [11]. Potential contributory factors to this higher rate of incidence among women include recurrent pregnancies and the fact that women predominantly engage in household and childcare activities. Additionally, we observed that patients with moderate and severe CTS had a significantly higher minimum F-wave latency than those with mild and normal CTS. A higher F latency value in milliseconds corresponds to an increased likelihood of CTS, suggesting that F-wave parameters are sensitive indicators of moderate and severe CTS.

Joshi et al. found that the minimum F latency is responsive to CTS, albeit less so than other electrophysiological parameters such as distal latency and sensory conduction velocity [9]. Likewise, Mondelli et al. highlighted that the F-wave is comparatively less sensitive than other parameters [10]. However, in these two studies, the patients were not classified into mild, moderate, or severe subgroups, and hence the parameters collected from mild or incipient CTS patients may have influenced the statistical results. Meanwhile, another study found that F-wave parameters are less susceptible to changes in mild CTS [12]. Therefore, in studies without subgrouping, the sensitivity of F parameters may be underestimated, emphasizing the need to create subgroups in future studies.

Although a previous study suggested that the minimum F-wave was sensitive to the disease, it did not identify at which point the sensitivity began, due to the absence of subgroup analysis (6). As per our understanding, three studies in the literature have demonstrated the sensitivity of F analysis while taking subgroups into account. Fisher and Hoffen presented a decrease in F velocity in 24 patients as the motor distal latency increased [13]. Aygül et al. found a significant delay in F latencies with progressive disease in their study of 57 patients [14]. Another comprehensive investigation into subgroups revealed a decrease in F-wave conduction velocity, correlated with disease severity [15]. However, the latter study lacked a detailed analysis of the precise stage at which sensitivity begins.

Our study, which analyzed electrophysiological parameters in a large group split into subgroups, revealed that F-wave slowing is highly sensitive to moderate and severe CTS. Moreover, the sensitivity of F-wave slowing increases as the disease severity worsens. Our study differs from previous studies as it incorporated the subgroup analysis in its methodology. Therefore, it is worth investigating the differences in F parameters between groups in clinical electrophysiology and its relationship with CTS complaints.

One possible limitation of our study is that, despite measuring minimum F-wave deceleration, we did not consider other parameters such as F-wave persistence, amplitude, and median-ulnar F comparison [16,17]. Further investigation of these F parameters will advance our comprehension of the electrophysiological features of CTS. In addition, the fact that the number of people in the severe CTS group was slightly lower than the other groups may have affected our statistical findings. Comparing people with moderate to severe CTS to age- and sex-matched controls will yield more comprehensive results.

## Conclusions

Our findings show that minimal F-wave latency is a reliable indicator of moderate to severe CTS. This sensitivity significantly increases in severe cases while disappearing in the presence of mild CTS. In light of this phenomenon, F parameters should be included in standard nerve conduction studies for the electrophysiological evaluation of CTS.

## References

[1] Chen JQ, Wang D, Liu B (2023). Body mass index and carpal tunnel syndrome: a case-control study. Medicine (Baltimore).

[2] Jiménez-Del-Barrio S, Cadellans-Arróniz A, Ceballos-Laita L, Estébanez-de-Miguel E, López-de-Celis C, Bueno-Gracia E, Pérez-Bellmunt A (2022). The effectiveness of manual therapy on pain, physical function, and nerve conduction studies in carpal tunnel syndrome patients: a systematic review and meta-analysis. Int Orthop.

[3] Panayiotopoulos CP, Chroni E (1996). F-waves in clinical neurophysiology: a review, methodological issues and overall value in peripheral neuropathies. Electroencephalogr Clin Neurophysiol.

[4] Rajabally YA, Varanasi S (2013). Practical electrodiagnostic value of F-wave studies in chronic inflammatory demyelinating polyneuropathy. Clin Neurophysiol.

[5] Lin CH, Tsai YH, Chang CH, Chen CM, Hsu HC, Wu CY, Hong CZ (2013). The comparison of multiple F-wave variable studies and magnetic resonance imaging examinations in the assessment of cervical radiculopathy. Am J Phys Med Rehabil.

[6] Hachisuka A, Komori T, Abe T, Hachisuka K (2015). Repeater F-waves are signs of motor unit pathology in polio survivors. Muscle Nerve.

[7] Curt A, Keck ME, Dietz V (1997). Clinical value of F-wave recordings in traumatic cervical spinal cord injury. Electroencephalogr Clin Neurophysiol.

[8] Sulaiman ME (2012). Appearance of F-wave during electrophysiological study of carpal tunnel syndrome. Tikrit J Pharm Sci.

[9] Joshi AG, Gargate AR (2013). Diagnostic utility of F waves in clinically diagnosed patients of carpal tunnel syndrome. Indian J Physiol Pharmacol.

[10] Mondelli M, Aretini A (2015). Low sensitivity of F-wave in the electrodiagnosis of carpal tunnel syndrome. J Electromyogr Kinesiol.

[11] Aboonq MS (2015). Pathophysiology of carpal tunnel syndrome. Neurosciences (Riyadh).

[12] Alemdar M (2015). Value of F-wave studies on the electrodiagnosis of carpal tunnel syndrome. Neuropsychiatr Dis Treat.

[13] Fisher MA, Hoffen B (1997). F-wave analysis in patients with carpal tunnel syndrome. Electromyogr Clin Neurophysiol.

[14] Aygül R, Kotan D, Ulvi H, Kuyucu M, Özdemir G, Ertekin A, Odabaş FÖ (2014). The relationship of median nerve F-wave parameters with severity and subtypes of carpal tunnel syndrome. J Back Musculoskelet Rehabil.

[15] El-Magzoub MS, Mustafa ME, Abdalla SF (2017). Neurophysiologic pattern and severity grading scale of carpal tunnel syndrome in Sudanese patients. J Neurol Neurosci.

[16] Uzunkulaoğlu A, Afsar SI, Tepeli B (2019). Terminal latency index, residual latency, and median-ulnar F-wave latency difference in carpal tunnel syndrome. Ann Indian Acad Neurol.

[17] Ginanneschi F, Mondelli M, Aretini A, Rossi A (2017). Reappraisal of the F/M amplitude ratio in carpal tunnel syndrome. Funct Neurol.

